# Statin use for cardiovascular disease prevention: perceptions among people living with HIV in the United States

**DOI:** 10.1186/s12875-024-02370-z

**Published:** 2024-04-17

**Authors:** Adedotun Ogunbajo, Ivy Todd, Deborah Zajdman, Abraelle Anderson, Soma Wali, Allison Diamant, Joseph A. Ladapo, Allison J. Ober

**Affiliations:** 1 RAND Corporation, Arlington, VA United States of America; 2 RAND Corporation, Pittsburgh, PA United States of America; 3 RAND Corporation, Santa Monica, VA United States of America; 4 Division of General Internal Medicine and Health Services Research, Department of Medicine, UCLA Geffen School of Medicine, Los Angeles, CA United States of America; 5 Department of Medicine, University of Florida College of Medicine, Gainesville, FL United States of America

**Keywords:** Cardiovascular disease, Statin therapy, People living with HIV, Social determinants of health

## Abstract

**Background:**

People living with HIV (PLWH) may be at heightened risk for cardiovascular disease (CVD). Statin use and lifestyle changes reduce the risk of CVD but remain under-prescribed among PLWH. The objective of this study was to characterize knowledge of CVD and statin use, current usage, barriers to taking statins, and information desired by PLWH to improve statin uptake among PLWH in Los Angeles, CA.

**Methods:**

Between April 2019 and April 2020, we conducted four focus group discussions (*n* = 37) with patients across three public community health clinics that serve PLWH in Los Angeles County, California. All clinics participated in a larger study to improve statin prescribing for PLWH. We asked about knowledge of statins, willingness to take a statin, possible barriers to statin usage, preferred information sources for health information, and desired information about statins. We utilized standard qualitative content analysis methods to identify themes.

**Results:**

We found a range in the awareness of statins, with some participants reporting never having heard of statins while others had a history of statin use. There were concerns about the potential long-term effect of statin use, but participants expressed willingness to use CVD medications generally and statins specifically, especially if recommended by their healthcare provider. Participants also expressed interest in potential alternatives to statin usage such as exercising regularly and nutritious eating.

**Conclusions:**

More interventions are needed to increase statin use among PLWH to improve CVD outcomes, which also has implications for HIV progression. Clinics should aim to increase patient and provider knowledge about CVD risk and statin use for PLWH and provide shared decision-making tools that are easy to use and culturally appropriate.

## Background

Since the introduction of effective antiretroviral therapy, people living with human immunodeficiency virus (PLWH) in the United States (U.S.) have experienced an increase in their life expectancy [1–3]. Consequently, with a longer lifespan, PLWH face increasing risk associated with older age including cardiovascular disease (CVD), which might be exacerbated by their existing HIV diagnosis and HIV medications. Past studies found that PLWH have an elevated risk for CVD (i.e. myocardial infarction, heart failure, and stroke) compared to people not living with HIV [4, 5]. Statins are prescription medications that lower cholesterol levels and reduce CVD risk, which can be highly beneficial for PLWH [6].

Statins have been demonstrated to slow the progression of CVD among PLWH on ART [7]. However, statin uptake among PLWH has been slow, with only 25–62% of PLWH clinically eligible for statins receiving the medication [8–11], and a limited amount of research describing factors that influence statin usage among PLWH has been conducted. A study exploring factors influencing CVD medication adherence (specifically medication to control hypertension and /or high cholesterol) among PLWH found: (1) discordance between patients’ strategies for CVD management and healthcare providers’ (HCP) recommendations (for example, some participants mentioned that their providers were more likely to prescribe them medications rather than strategies for lifestyle modification, which was their preference), (2) respondents used their experience with ART medication as a reference point for guidance on CVD prevention, leading to sustainable adherence behaviors, and (3) multidimensional (individual, interpersonal and community level) motivations for CVD medication adherence that ranged from an internal sense of accomplishment to social connections with family, friends and HCPs [12]. A recently published study conducted by our group and from the parent grant, of various HCPs (physicians, nurse practitioners, registered nurses, and physician assistants) providing services to PLWH found that while CVD risk assessment for PLWH was considered standard medical practice, various patient factors such as critical health needs, substance use, mental health, and other socioeconomic barriers often take priority over CVD risk assessment and prescription of statins, given short appointment times and acuity of these other needs [13]. Similarly, another study of HCPs, specifically HIV specialists and cardiologists, found that barriers to referring PLWH to CVD care included lack and cost of transportation, insurance coverage gaps, stigma, and patient reluctance [14]. While informative, prior studies do not address barriers, facilitators, and motivators to starting statin from the perspective of PLWH. Understanding these factors is critical to improving the utilization of statins among PLWH and informing CVD care management protocols among HCP who provide services to PLWH.

The objective of this study was to characterize knowledge of CVD & statin use, current usage, barriers to taking statins, and information desired by PLWH to improve statin uptake. A primary goal of the study was to use the data to create a patient education brochure based on patient needs.

## Methods

### Study setting

Between April 2019 and April 2020, we conducted four focus group discussions (*n* = 37) with patients across three of eight community health clinics that serve PLWH in Los Angeles County, California participating in a larger study (INSPIRE) to improve statin prescribing for PLWH [15]. The participating clinics fall into three categories: federally qualified health centers (FQHC) (*N* = 1); non-FQHC community clinics (*N* = 1), and community ambulatory care clinic (CACC) within a hospital setting (*N* = 1). All clinics serve a racially/ethnically diverse population of PLWH, focus on the care of underserved populations, and include a mix of clinicians providing primary care services including infectious disease specialists, primary care physicians with and without an HIV specialization, nurse practitioners, and physician assistants. Three focus groups were conducted in English, and one was conducted in Spanish.

This study was approved by the University of California, Los Angeles Institutional Review Board. All participants provided verbal consent to participate. All study procedures were carried out by relevant guidelines and regulations.

### Procedures

We conducted four focus groups with patients across three participating clinics in the INSPIRE study. Patients who met the following inclusion criteria: (a) 40 years or older, (b) having been diagnosed with HIV, and (c) willing to participate in an interview with other participants, were provided information by clinic staff about participating in the focus group. The focus group discussions lasted approximately an hour. Participants were offered a $50 gift card for participating in the focus group. Focus groups were recorded and transcribed. Participants were reminded of study procedures and provided informed verbal consent to participate and be recorded.

### Measures

The interview guide covered the following topical areas: knowledge of statins, including knowledge about common health problems related to cardiovascular disease (e.g., high cholesterol, high blood pressure, diabetes); risk factors associated with heart health (e.g., smoking, substance use, stress); CVD risk prevention and ways to lower risk, including statins and lifestyle habits; willingness to take a statin; and preferred information sources for health information and desired information about statins. Initial questions were broad and open-ended and were followed up with closed-ended questions to clarify responses and obtain greater understanding and insight.

### Analysis

The research team (investigators AJO, AO; research assistants (IT, DZ) read all transcripts and developed the codebook based on broad categories that emerged: Knowledge about cardiovascular disease and statins; desired information and willingness to take statins; and barriers to statin use. The team discussed and finalized subdomains of emergent themes. Using Dedoose (a qualitative analysis software program), the team first entered primary and subdomain themes into the codebook. IT and DZ then marked areas of text about each domain and construct code. IT and DZ started with a random sample of 20% of transcript sections, coding independently and reviewing together. If coder disagreement revealed ambiguity in the codebook, we modified the codebook. Training continued until the two coders could consistently identify and mark each theme. Next, both coders each worked on two interviews independently, after which measured coder consistency was assessed. Once consistency was reached, evidenced by Kappa of ≥ 0.70 considered “good” consistency [16], the remainder of the transcripts were coded independently. Themes that did not fall into one of the codebook domains were marked as “other.” The analysis team categorized these themes and added them to the codebook. The research assistants then marked text about these codes.

## Results

### Patients lack knowledge of CVD and associated risk for PLWH

Participants in all four groups demonstrated an overall lack of knowledge or accurate understanding of CVD. There appeared to be a lack of general knowledge of how CVD affects bodily functioning:

*“I never knew the differences between a good and a bad heart health, I just thought it’s either you have high cholesterol or low cholesterol” FG 3*.

Additionally, some participants admitted their lack of knowledge about CVD and awareness of risk:

*“I’m not ready for information about heart disease because there’s nothing wrong with me in that area. Even though I have had the virus [HIV] for 30 years I never thought about no heart disease.”* FG 1.

When asked about how CVD may be related to HIV, participants demonstrated some knowledge of the relationship between CVD and HIV. Specifically, there was agreement that HIV reduced the immune capability of the body in fighting other diseases, which they believed could lead to an increased risk of CVD. One participant stated:

*“I understand that HIV is a virus that attacks the immune system of the individual. Therefore, the people who have this virus are at high risk not only of having heart disease but a lot of other diseases.”* FG 2.

Conversely, some participants lacked knowledge of any link between how living and aging with HIV could exacerbate their risk for CVD:

“*Thinking that heart problems are just going to just materialize from your HIV infection, I just never heard of that.”* FG 3.

### There was a range of awareness of statin use to lower the risk for heart problems among PLWH

Awareness of statin use to lower risk for heart problems ranged vastly among participants with some participants reporting having never heard of statins, while others had a history of statin use. One participant stated:

*“I never had high cholesterol, so I don’t know nothing about statins.”* FG 4.

Conversely, some participants reported a history of statin use—implying knowledge of statin—but with varied results. One participant described seeing their cholesterol levels reduce dramatically as a result of being prescribed a statin by their HCP:

“*My cholesterol got a little over the high mark and my HIV doctor didn’t present it to me in an HIV picture. She just said, ‘your cholesterol is high’ – it wasn’t HIV or your medication causing it, it was ‘your cholesterol was high’. And she put me on statins and my cholesterol has dropped to one-half of what it was. It was 210 and it went to 91.”* FG 1.

Another participant described low-dosage statin use but with suboptimal adherence after their cholesterol numbers were reduced. They also noted no active monitoring of statin use by health providers, which contributed to an even further lack of prioritization:

*“My cholesterol is crazy and that’s something that’s been developing ever since they started me on antiretrovirals; they started to see numbers in my cholesterol that they were concerned with. So, I’ve been taking a statin drug for the last couple decades, the lowest dosage, and to be honest with you sometimes I would go for a month or so where I just never got around to taking it before I fell asleep because it was the last thing I would take at night. But it never showed any change on the very low cholesterol numbers that I had. So, it was one of those drugs where if I didn’t take it, it didn’t show up in the bloodwork, nobody cared, and I got to be sort of lazy.”* FG 3.

### Patients are interested in information about statins and have concerns about side effects and contraindications

Most focus group participants were interested in general information about statins. Participants were interested in how statins might affect the body, how often to take them, and other pertinent logistical information. Two groups mentioned concern over the side effects of statins. One participant suggested:

*“Be more precise about what they’re [HCP] recommending and issuing to us. Be more specific about what the medication that we’re taking, how would it affect us. Because we don’t have much, we don’t have much reading up on this stuff. To tell you the truth, I don’t even know what all the pills are made of or what consists inside the pill. I know damaging as it be to the heart or the kidney because those two major points play in HIV for me. So, I need to know more about it.” FG 2*.

Participants also expressed willingness to use CVD medications generally and statins specifically, especially if recommended by their HCP. This participant described being willing to utilize heart medicine with plans to monitor (through laboratory testing) its efficacy and deciding whether to stay on the medication based on the lab results:

*“If I was suggested to take a heart medicine I would try it and see how it mixes with everything else I was taking for two weeks; and then go back to the doctor, get my blood drawn again and see how it’s working. And if it’s working, great, I’ll stay on it; and if it’s not working, I’ll stop taking it.”* FG 1.

Another participant recognized the potential for a decrease in healthy heart function, especially as they age, and expressed being acceptable to physician recommendations for medications to improve heart health:

*“Well, if the doctor suggests taking some medication to make my heart stronger, well, that’s great. Your heart doesn’t work like a 20-year-old heart, your heart also gets tired, so, if the doctor suggests I take some medication to strengthen my heart, that will be great. I would just ask him if that medication has very bad side effects for your health, in general, but that’s it. But I would take it, of course I would.”* FG 4.

### Concern about side effects impedes statin usage

Participants expressed hesitation and ambivalence towards statin use due to concern about side effects, and past experiences with HIV medications. One participant expressed concerns about the possible immediate side effects of the additional drug given existing medications:

*“It’s a win and a lose situation because the pill may be good but then you’re adding something else to what you got going already, and you don’t know the side effect from that pill.”* FG 2.

Another participant expressed concerns about potential long-term effects of statin use such as organ damage but also recognized the possible inevitability of an early death if the medication was not taken:

*“The truth is that medication, in the long run, damages certain organs; but what can you do if you need it? Even if it’s damaging you inside, we have to take it even if we don’t want to because otherwise, we die or we don’t feel well, so, it’s mandatory, you have to take it even if you know it’s affecting our system.”* FG 1.

### Patients are interested in trying alternatives to statins based on lifestyle modifications

Several participants expressed an interest in utilizing alternative strategies to prevention and maintaining better heart health and cholesterol levels that did not include medication usage. Specifically, they mentioned preferring a holistic approach that included more exercising, consuming antioxidants, and stopping alcohol consumption. They viewed this approach as healthier and could help guard against medication addiction:

*“The extra little exercise and the extra little antioxidants that she told me to eat brought my cholesterol way back to normal even without a pill. I’m in recovery too, almost two years clean and sober and I’m against pills. I’m getting ready to have a surgery on my knee and I won’t take nothing for it because I know if I start taking it I’m going to want to take more and more and more because I know it’s gonna decrease the pain. But I’m willing to accept that little extra pain to stay a little healthier. Because I know I won’t be as healthy as I am now if I was to take those extra pills.”* FG 3.

Another participant described focus on maintaining a healthier diet and how that helped reduce their blood pressure in the past:

*“I’m doing it all through my diet. I had high blood pressure at one point but through my diet I’ve dropped my blood pressure. Recently, I was still taking the high blood pressure medication but they’re like, ‘You don’t really have a problem with the high blood pressure anymore, do you want to stop?’ Sure, please.”* FG 4.

Another participant stated:

*“I’m trying to maintain it [cardiovascular health] with diet and everything like that. I don’t really rely on drugs as much as I try to you know, put healthy things in and try and you know… Because like say for instance the drugs, when I research the drugs, when I find out this one that’s doing this could have these side effects, I’m, ‘Okay look, can we stop this one? Can we try to take something over here so then I don’t have to worry about the liver and the kidneys’”* FG 2.

## Discussion

This study examined knowledge of CVD and statin use, current usage, information desired by PLWH, and barriers to taking statins. We found a range in the awareness of statin use, with some participants reporting never having heard of statins while others had a history of statin use. However, participants expressed willingness to use CVD medications generally and statins specifically, especially if recommended by their HCP, which is similar to findings from our provider-focused study in which providers expressed that, despite some hesitation, if providers recommend statins to their patients through shared decision-making, patients generally comply [13]. While there were some concerns about potential long-term adverse effects of statin use, participants expressed interests in alternatives to statin usage such as adopting exercising and healthy eating. This finding also was consistent with the sentiments of providers [13]. These findings reinforce the need for interventions and programs specifically designed to increase uptake of preventive behaviors, increase knowledge of statins, and promote conversations between HCPs and patients, with the goal of increasing healthy habits and statin utilization among PLWH who meet clinical criteria.

Our findings of variability in patient awareness and knowledge of statins is consistent with a previous study of HCPs who also expressed their own knowledge gaps related to statin prescription for PLWH [13, 17]. Importantly, another study found higher adherence to statin among PLHIV compared to people who were HIV negative and HIV status did not affect statin-risk perception [18]. It is possible that lack of provider knowledge might be associated with lack of patient knowledge and under prescription of statins for statin-eligible PLWH [19]. Consequently, it is important that HCPs who provide services to PLWH receive training on the CVD risk among PLWH generally and potential benefits of prescribing statin to their eligible patients. A systematic review examining patient oriented interventions aimed at increasing statin-prescribing rates among the general population found that patient education initiatives were the most effective and physician education programs without education for patients was the least effective [20]. As such, developing and disseminating patient-friendly educational materials on the utility and benefits of statin usage that is tailored to the unique lived experiences of PLWH may improve uptake of statins (example brochure shown in Appendix). A major challenge to providing CVD care to PLWH is that these patients may receive services primarily from infectious disease specialists, whose primary focus typically is on HIV treatment and management, not CVD. Consequently, PLWH may benefit from having a primary care provider in addition to an infectious disease specialist. Also, HCPs can engage in shared decision-making and can incorporate educational materials into those discussions. Shared decision-making is a process in which clinicians and clients work together to make decisions and select tests, treatments, and care plans based on clinical evidence in a manner that balances risks and expected outcomes with client preferences and values [21, 22]. These strategies might help increase statin knowledge among PLWH and willingness to take statins.

Interestingly, while there was some limited prior knowledge of statins among many participants, they expressed an overall willingness to use statins. This is especially important as there is evidence demonstrating that statin use lowers low-density lipoprotein (LDL) and oxidized LDL cholesterol among PLWH [23]. There is also preliminary evidence of possible CVD morbidity and all-cause mortality benefits for PLWH [23]. Participants expressed willingness to take a statin if it were recommended by their HCP, providing more evidence for the need for patient-provider communication and shared decision making around statin utilization. Additionally, participants raised some concerns around potential side effects and long-term effects of statin usage for PLWH. While research has shown that statins generally have few side effects within the context of antiretroviral medication [23], it is still imperative that providers provide patients with information about potential side effects and risk associated with statin utilization to facilitate informed decision making.

There are some study limitations to consider. First, this was a small convenience sample of PLWH at three clinics and therefore not representative of the lived experiences of all PLWH, reducing the generalizability of the study findings. Second, while we included only patients 40 years or older in the sample (statins typically are not given to those in age groups younger than 40), we did not ascertain additional CVD risk factors, thus participants in the sample were not necessarily those for whom a statin would be indicated. Nevertheless, the sample was drawn from three different types of clinics and included English and Spanish speakers, and we found common themes across all groups.

## Conclusion

This study examined knowledge of CVD and statin use, current usage, information desired by PLWH, and barriers to taking statins. We found a range in the awareness of statin use, with some participants reporting having never heard of statins while others had a history of statin use. While there were some concerns about potential long-term side effects of statin use, participants generally expressed willingness to use statins, especially if the medication was recommended by their clinicians. Of note, some participants expressed interest in alternatives to statin usage, such as participating in regular physical activity and nutritious eating. More interventions are needed to increase preventive cardiovascular behaviors and clinically appropriate statin use among PLWH to improve CVD outcomes, which also has implications for HIV progression. Healthcare centers should aim to increase both patient and provider knowledge about CVD risk and statin use for PLWH and provide shared decision-making tools that are easy to use and culturally appropriate.

## Data Availability

The datasets generated and/or analyzed during the current study are not publicly available due the sensitivity of the topic but are available from the corresponding author on reasonable request.

## Abbreviations

PLWH: People living with human immunodeficiency virus
CVD: Cardiovascular disease
HCP: Healthcare providers
ARTs: Antiretroviral therapy
